# Biomechanically, structurally and functionally meticulously tailored polycaprolactone/silk fibroin scaffold for meniscus regeneration

**DOI:** 10.7150/thno.44270

**Published:** 2020-04-06

**Authors:** Zong Li, Nier Wu, Jin Cheng, Muyang Sun, Peng Yang, Fengyuan Zhao, Jiahao Zhang, Xiaoning Duan, Xin Fu, Jiying Zhang, Xiaoqing Hu, Haifeng Chen, Yingfang Ao

**Affiliations:** 1 Institute of Sports Medicine, Beijing Key Laboratory of Sports Injuries, Peking University Third Hospital, 49 North Garden Road, Haidian District, Beijing 100191, People's Republic of China; 2 Department of Biomedical Engineering, College of Engineering, Peking University, 5 Yiheyuan Road, Haidian District, Beijing 100871, People's Republic of China

**Keywords:** 3D printing, SMSC-specific affinity peptide, meniscus repair, polycaprolactone, silk fibroin

## Abstract

Meniscus deficiency, the most common and refractory disease in human knee joints, often progresses to osteoarthritis (OA) due to abnormal biomechanical distribution and articular cartilage abrasion. However, due to its anisotropic spatial architecture, complex biomechanical microenvironment, and limited vascularity, meniscus repair remains a challenge for clinicians and researchers worldwide. In this study, we developed a 3D printing-based biomimetic and composite tissue-engineered meniscus scaffold consisting of polycaprolactone (PCL)/silk fibroin (SF) with extraordinary biomechanical properties and biocompatibility. We hypothesized that the meticulously tailored composite scaffold could enhance meniscus regeneration and cartilage protection.

**Methods**: The physical property of the scaffold was characterized by scanning electron microscopy (SEM) observation, degradation test, frictional force of interface assessment, biomechanical testing, and fourier transform infrared (FTIR) spectroscopy analysis. To verify the biocompatibility of the scaffold, the viability, morphology, proliferation, differentiation, and extracellular matrix (ECM) production of synovium-derived mesenchymal stem cell (SMSC) on the scaffolds were assessed by LIVE/DEAD staining, alamarBlue assay, ELISA analysis, and qRT-PCR. The recruitment ability of SMSC was tested by dual labeling with CD29 and CD90 by confocal microscope at 1 week after implantation. The functionalized hybrid scaffold was then implanted into the meniscus defects on rabbit knee joint for meniscus regeneration, comparing with the Blank group (no scaffold) and PS group. The regenerated meniscus tissue was evaluated by histological and immunohistochemistry staining, and biomechanical test. Macroscopic and histological scoring was performed to assess the outcome of meniscus regeneration and cartilage protection *in vivo*.

**Results**: The combination of SF and PCL could greatly balance the biomechanical properties and degradation rate to match the native meniscus. SF sponge, characterized by fine elasticity and low interfacial shear force, enhanced energy absorption capacity of the meniscus and improved chondroprotection. The SMSC-specific affinity peptide (LTHPRWP; L7) was conjugated to the scaffold to further increase the recruitment and retention of endogenous SMSCs. This meticulously tailored scaffold displayed superior biomechanics, structure, and function, creating a favorable microenvironment for SMSC proliferation, differentiation, and extracellular matrix (ECM) production. After 24 weeks of implantation, the histological assessment, biochemical contents, and biomechanical properties demonstrated that the polycaprolactone/silk fibroin-L7 (PS-L7) group was close to the native meniscus group, showing significantly better cartilage protection than the PS group.

**Conclusion**: This tissue engineering scaffold could greatly strengthen meniscus regeneration and chondroprotection. Compared with traditional cell-based therapies, the meniscus tissue engineering approach with advantages of one-step operation and reduced cost has a promising potential for future clinical and translational studies.

## Introduction

A meniscus injury is a common musculoskeletal disease in knee joints, which can cause abnormal stress distribution, reduced lubrication, instability, and subsequent cartilage degeneration, ultimately leading to progressive osteoarthritis 1. As a result of inadequate blood supply to the inner two-thirds of the meniscus, injuries are often unable to heal without surgical intervention. The current treatment strategy mainly includes meniscus suture, partial or total meniscectomy, and allograft transplantation. Although suture and meniscectomy delay the degeneration of articular cartilage, they cannot prevent osteoarthritis. As an alternative treatment, meniscus allograft transplantation had some potential; however, its long-term results were still dissatisfactory and uncertain 2. The commercialized meniscal implants, such as CMI^®^, Actifit^®^, and NUsurface^®^, have been applied in the clinic, but there are several questions about implant shrinkage, non-degradation, and uncertain long-term effects. Also, none of the commercial implants can perfectly restore or permanently replace the natural meniscus tissue, effectively solve the symptoms after meniscectomy, and prevent degenerative cartilage diseases 3. In complex meniscus injuries, the inability of surgical intervention to recover the structural, biomechanical, and functional properties of meniscus remains a great challenge.

Recent advances in tissue engineering may provide a promising treatment for meniscus repair 4. Kim et al. implanted an alginate gel scaffold to repair meniscus defects in rabbits 5. Although the neo-meniscus had similar histology structure and biochemical content, the tensile modulus of implants was inferior to the native meniscus. Mandal et al. used multilayered silk scaffolds for meniscus tissue engineering, which showed good cell infiltration and ingrowth, but the biomechanical properties of regenerated meniscus were far inferior to the native meniscus 6. These studies have investigated several different tissue engineering scaffolds for total meniscus reconstruction. Although these scaffolds exhibited favorable biocompatibility *in vitro* and *in vivo*, their biomechanical properties were not comparable to those of the native meniscus 6, 7. Therefore, scaffolds with excellent biocompatibility and biomechanics should be further investigated.

In our previous study 8, a silk fibroin (SF) cartilage tissue engineering scaffold with optimized structure and function was constructed that exerted good repair effects. Since the meniscus SF scaffolds could not achieve the required biomechanical properties *in vivo*, we introduced a widely applied polyester polymer material polycaprolactone (PCL), which has good printability and superior biomechanical properties 9, 10. The spatial structure of biomimetics has been shown to be a key factor for mesenchymal stem cell (MSC) proliferation and differentiation. The biomimetic architecture provided by 3D printing (3DP) technology has a relatively high similarity to the native meniscus and allow individualized size parameters in comparison with traditional electrospinning preparation methods 11. Although PCL has been used extensively in 3DP in previous studies, the utilization of 3D printed PCL to reinforce the mechanical properties is seldom. Pure PCL scaffolds could provide robust mechanical strength and biomimetic spatial structure but have the risk of attrition of articular cartilage and lack of biological functional bionics. Also, to avoid the abrasion of the articular cartilage of pure rigid PCL scaffold, the addition of elastic SF sponge on the PCL scaffold could improve the capacity of shock absorption and reduce the frictional force of interface between the scaffold and articular cartilage. With a slow degradation, the PCL scaffold could withstand biomechanical support during the period of tissue ingrowth and remodeling.

It has been reported that exogenous MSCs seeded on scaffolds improved the repair efficiency of meniscus 10, 12; however, other issues, such as immune rejection response, disease transmission risk, and the difficulty of cell expansion, obstructed the clinical application of MSCs 13. Hence, it is imperative to investigate materials with excellent biomechanics and the ability to improve endogenous MSC recruitment and retention of the scaffolds by functional modification and, therefore, might be better suited for future clinical translation. To improve MSC-specific homing, we had identified a synovium-derived mesenchymal stem cell (SMSC) affinity peptide (LTHPRWP; L7) through phage display technology, which displayed specific recruitment to SMSCs both *in vitro* or *in vivo* 14. After functional modification of the scaffold, it could build up a preferable microenvironment for cell growth and differentiation.

In the present study, we used silk fibroin (SF), polycaprolactone (PCL), and a synovium-derived mesenchymal stem cell (SMSC)-specific affinity peptide and constructed a biomechanically, structurally, and functionally tailored tissue engineering scaffold through 3DP. The meticulously designed scaffold exhibited better autologous MSC recruitment, differentiation, meniscus regeneration, and chondroprotection.

## Methods

### Preparation of the scaffolds

**Figure 1:**
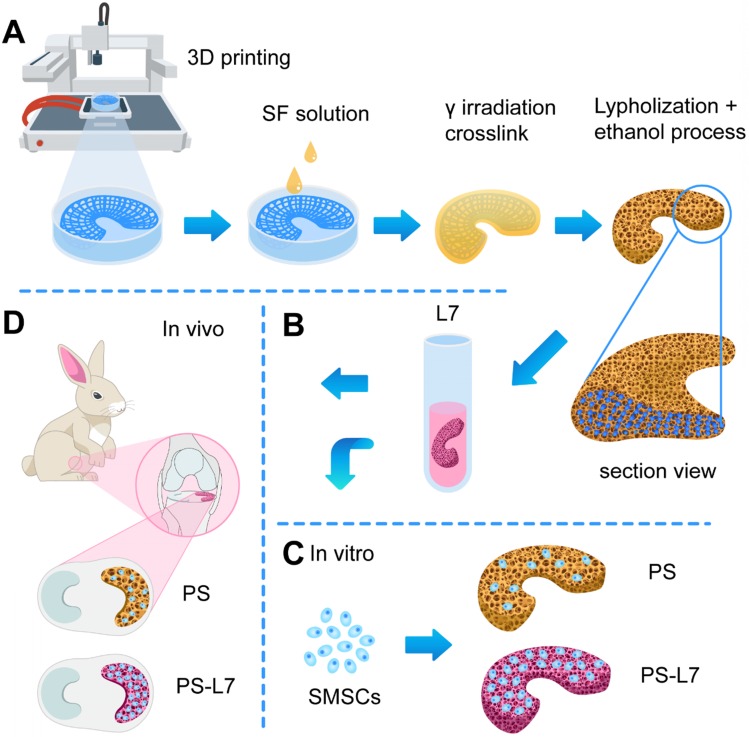
Schematic illustration. (A) Fabrication and crosslinking of the scaffolds. (B) Functional optimization of the scaffolds. (C) Biocompatibility assessment *in vitro*. (D) Implantation *in vivo*.

The biomimetic meniscus scaffolds were fabricated using a 3D-Bioplotter (EnvisionTEC 4th generation) (**Figure 1A**). Briefly, we measured anatomical parameters of the rabbit medial meniscus, included, anterior and posterior horn widths, anterior and posterior horn lengths, posterior horn to anterior horn distance, body width, and body thickness. A 3D computer-aided design (CAD) medial meniscus model of a wedge-shaped arc disk was designed via SolidWorks (Autodesk, USA). PCL (average molecular weight of 80,000 g/mol) was prepared in the high-temperature cartridge and melted at 130°C for 30 min. Subsequently, PCL was extruded through a 300-µm diameter nozzle pneumatically at the pressure of 0.8 MPa and the speed of 7.0 mm/s, following the predefined internal bionic microstructure. A 300-µm microchannel was determined to be optimal to approximate the biomechanical properties of the native meniscus.

### Preparation of SF solution

Purified SF was prepared by removing the glue-like protein sericin from the cocoons in a 0.02 M boiling sodium carbonate solution for 1 h, followed by rinsing with distilled water to remove the degumming solution. A 9.3 M lithium bromide solution was used to dissolve the purified SF for 1 h at 70°C and dialyzed in distilled water for 48 h using benzoylated dialysis tubing (MWCO: 2 kDa). SF was concentrated against a 20 wt% poly (ethylene glycol) solution for at least 6 h. The final concentration of SF was determined by measuring the dry weight of the SF solution placed in the oven at 70°C overnight. The prepared SF solution was stored at 4°C until use 15.

### Crosslinking

The PCL scaffolds were immersed in the SF solution and irradiated with γ-rays from a Co-60 source at 75 kGy at room temperature 16. Next, the scaffolds were pre-frozen at - 20°C overnight and freeze-dried for 48 h. The scaffolds were then secondarily cross-linked in ethanol overnight, washed repeatedly and thoroughly in PBS pre-frozen and freeze-dried again, and then stored at -20°C until use.

### Characterization of scaffolds

#### Scanning electron microscopy (SEM)

**Figure 2:**
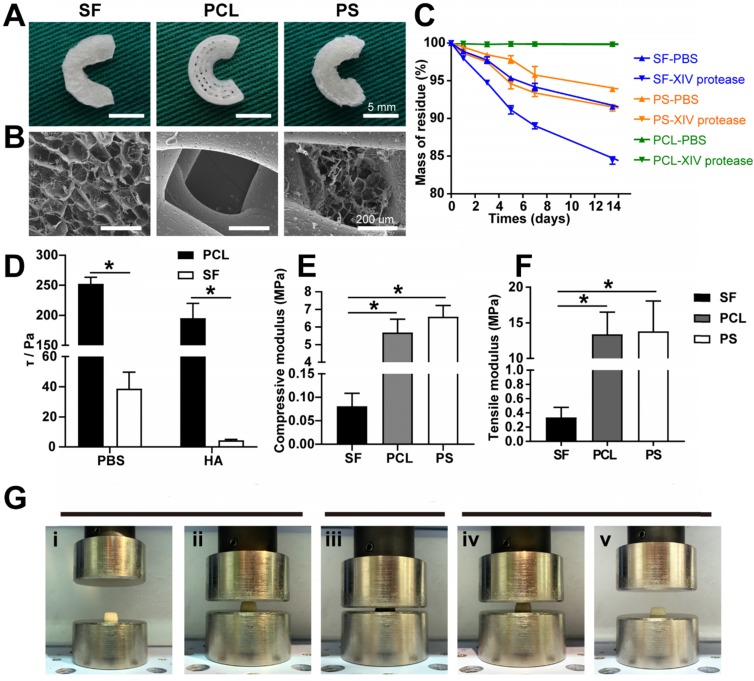
Scaffold characterization. (A) Macroscopic observation of the scaffolds. (B) SEM images of the scaffolds. (C) Degradation rate of the scaffolds. (D) Interfacial shear force of the scaffolds in PBS and HA. (E) Compressive modulus of the scaffolds *in vitro*. (F) Tensile modulus of the scaffolds *in vitro* (n = 4, *p < 0.05). (G) Elastic detection of the SF scaffold showing good memory-shape characteristic and elasticity: (i+ii) initial appearance of the SF scaffold before compression, (iii) scaffolds under a compressive strain of 80%, (iv+v) returning to the original shape.

The scaffolds were fixed with 2.5% (v/v) glutaraldehyde buffered with PBS, dehydrated using a graded series of ethanol washes, and dried to a critical point (EM CPD300; Leica, Wetzlar, Germany) using carbon dioxide (CO_2_). Samples were sputter-coated with gold prior to SEM observation. The pore size and microstructure of the scaffolds were observed using SEM (S-4800 field emission scanning electron microscope; Hitachi, Tokyo, Japan) (**Figure 2B**).

#### Degradation *in vitro*

The three kinds of scaffolds were immersed in PBS (pH = 7.4) and XIV protease solution (0.5 U/mL) and incubated at 37°C (**Figure 2C**). At day 1, 3, 5, 7, and 14, the scaffolds were weighed with a microbalance after removing excess water (Wt). The initial mass of scaffolds was recorded as W0. The degradation ratio was calculated as 100% × (W0 - Wt)/W0.

#### Frictional force of interface

The scaffold (n = 5 each) was punched to form disks of 10 mm in diameter, and the frictional force of the scaffold was measured in PBS or hyaluronic acid (HA, 1 mg/mL 17) at 37°C utilizing a rheometer (ARES-G2, TA Instruments, America) as described previously (Fig. 2d) 18. Briefly, samples were pre-hydrated in PBS or HA in a water bath at 37°C for 24 h prior to testing. Subsequently, the upper coaxial disk-shaped platen was covered by a piece of smooth glass, and the lower platen was glued to the scaffold. After that, the interface between the sample and glass was immersed in PBS or HA at 37°C. Then, at a strain-constant mode, the lower platen was rotated with an angular velocity, and the torque and normal force were recorded.

#### Biomechanical testing

Scaffold specimens of different sizes (10×10×10 mm cubes for unconfined compressive strength and 5×5×15 mm for tensile strength) were prepared for biomechanical testing using an AGS-X Precision Universal Tester (SHIMADZU, Japan) (**Figure 2E-F**). All specimens were kept moist using PBS throughout these tests.

#### Fourier transform infrared (FTIR) spectroscopy analysis

**Figure 3:**
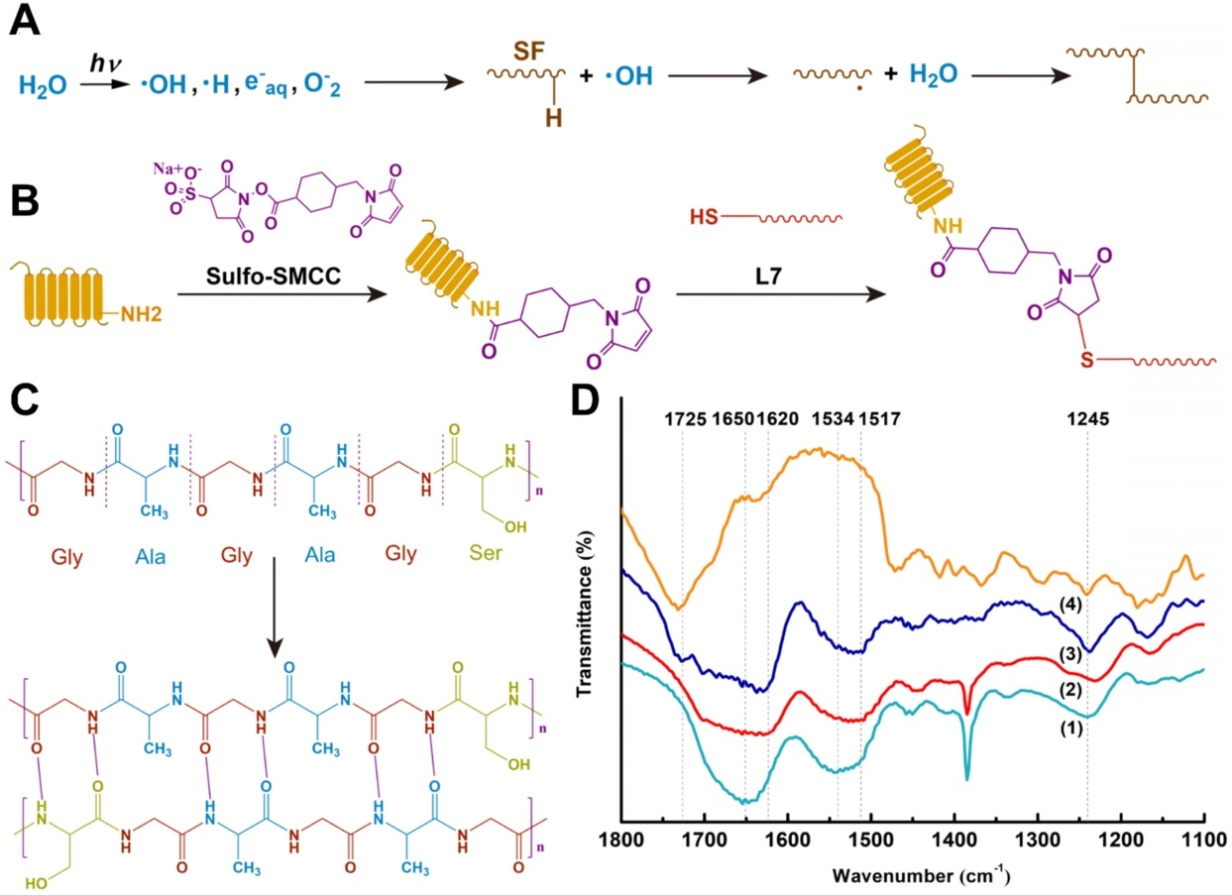
Crosslinking procedures of the scaffolds. (A) Proposed cross-linking mechanism of SF under γ-irradiation. (B) Conjugation of L7 peptide onto the scaffolds. (C) Secondary structure changes of SF after ethanol treatment. (D) FTIR spectra of the samples: (1) Pure silk solution, (2) SF after γ-crosslinking and ethanol processing, (3) SF-PCL after γ-crosslinking and ethanol processing, (4) Untreated PCL.

To analyze the scaffold structure and crosslinking effect, PS scaffolds were lyophilized and crushed to powder form. FTIR spectrometry was performed on an Affinity-1S FTIR spectrometer using the KBr disk technique from 4000 to 400 cm^-1^ (**Figure 3D**). The resolution was 4 cm^-1^ and the scanning frequency was 5 times.

### Functional modification of scaffolds

A proven SMSC affinity peptide L7 (LTHPRWP, molecular weight 1009.21 Da) 14 was commercially synthesized and purified (Scilight-Peptide Inc., Beijing, China). To facilitate conjugation to the scaffolds or fluorescein-5-isothiocyanate (FITC)-labeling, an extra cysteine was linked to the carboxyl terminus. The peptide conjugation was performed as previously described (**Figure 3C**) 19. Briefly, the PS scaffolds were immersed in 1 mL of 10% (w/v) 1,6- hexanediamine solution for 1 h at 37°C. Subsequently, the scaffolds were gently washed twice with ultrapure water and soaked in 400 µl of sulfosuccinimidyl 4-N-maleimidomethyl cyclohexane-1-carboxylate (Sulfo-SMCC, Thermo Fisher Scientific Inc., USA) solution (2 mg/mL) for 1 h at room temperature. The PS scaffolds were then incubated in 4 mL of L7 peptide solution (0.1 mg/mL) for 24 h at 4°C. Ultimately, the PS-L7 and PS scaffolds were freeze-dried, sterilized with cobalt-60 for 24 h, and stored at -20°C before use.

### Biocompatibility of scaffolds

#### SMSC isolation and culture

SMSCs were isolated and cultured as previously described with slight modification 20. Synovial tissue was harvested from the suprapatellar pouch of 80 g Sprague-Dawley (SD) rats, which overlays the non-cartilaginous areas of the femur through an arthrotomy of the knee. Synovial tissues were washed three times with PBS and then digested with 0.2% collagenase type I (Invitrogen, Carlsbad, CA, USA) in α-MEM for 30 min at 37°C. The released cells were suspended in complete medium. The fresh medium was replaced every 3 days. After 90% confluence was reached, the cells were diluted (1:3) and passage 3 SMSCs were used for the cytocompatibility studies.

#### SMSC seeding on the scaffolds

The scaffolds were sterilized with cobalt-60 for 24 h and by UV light for 1 h before use in a laminar flow bench for cell seeding. Each scaffold was seeded with 1×10^6^ SMSCs in 50 µL medium (DMEM + 10% FBS). Cells were allowed to adhere for 2 h; then, fresh medium was added and changed every 3 days.

#### *In vitro* recruitment capacity and cell viability

**Figure 4:**
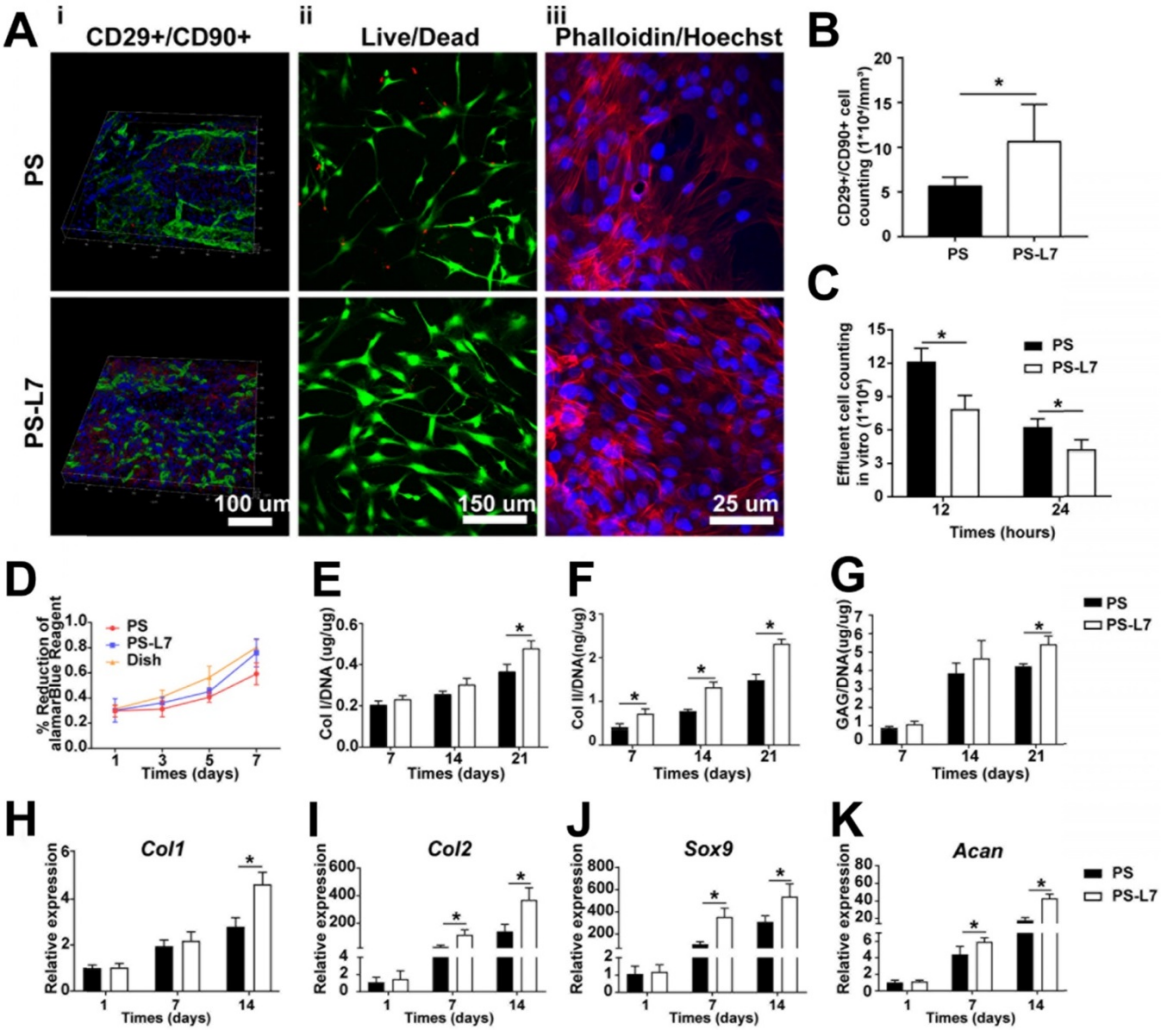
Biocompatibility, recruitment, and chondrogenic differentiation of SMSCs in the scaffolds* in vitro* and *in vivo*. (A) i) SMSC recruitment was verified using immunofluorescence assay after 1-week of implantation with different scaffolds *in vivo*. ii) Viability of SMSCs was analyzed by Live/Dead staining 3 days after seeding on different scaffolds without chondrogenic incubation. iii) Morphology of SMSCs was observed via Phalloidin/Hoechst assay after 3 days of culturing with different scaffolds without chondrogenic induction. (B) Number of CD29+/CD90+ double-positive cells on different scaffolds *in vivo* at 1-week post-surgery. (C) Number of effluent cells at 12 and 24 hours after SMSCs were seeded on different scaffolds *in vitro*. (D) Viability of SMSCs in different groups was observed by alamarBlue assay, and the OD value at each point was normalized against the average of the first day in each group. No significant difference among different groups was observed at the same time point. (E-G) Cartilaginous matrix production in different scaffolds: (E-F) Col I and Col II production quantified by ELISA; (G) GAG assay. (H-K) cartilage-specific gene expression of Col I, Col II, Sox 9, and ACAN (n = 6, *p < 0.05).

To confirm specific cell recruitment capacity of the L7 peptide, the effluent of cells was counted 12 h and 24 h after SMSCs were implanted on scaffolds (n = 6). After 3 days of culture, the SMSCs on different scaffolds were analyzed with LIVE/DEAD staining (Invitrogen, Carlsbad, CA, USA) using confocal microscopy to assay the viability of SMSCs on the scaffolds (**Figure 4Aii**). Briefly, the cells were immersed in 1 mL of a working solution containing 2 mM calcein-AM and 4 mM ethidium homodimer-1 reagents followed by incubation at room temperature for 1 h. Excitation wavelengths of 488 nm or 568 nm were used to detect the visualization of calcein AM (green fluorescence: labeling live cells) or ethidium homodimer-1 (red fluorescence: labeling dead cells). SMSC viability on different scaffolds was quantified by the AlamarBlue assay (Thermo Scientific, USA) (n = 5), with cells cultured in a dish as a positive control (**Figure 4D**). After 1, 3, 5, and 7 days of culture, 50 μL of alamarBlue solution was added to the medium and cultured for 4 h at 37°C. The OD value was then measured at 570 nm using a Varioskan Flash reader.

#### Morphology of SMSCs on the scaffolds

The morphology of SMSCs on both scaffolds was observed under confocal microscopy (Leica, Nussloch, Germany) (**Figure 4Aiii**). Briefly, the two SMSC-loaded scaffolds were washed with PBS three times each for 5 min and then fixed with 4% paraformaldehyde for 30 min at room temperature. Rhodamine-Phalloidin (160 nM; Cytoskeleton Inc., Denver, CO, USA) was used to stain the cytoskeleton of the SMSCs for 1 h at 37°C. After washing three times, the nuclei were counterstained with Hoechst33258 (2 μg/mL; Fanbo, Beijing, China) for 10 min.

#### Biochemical assays for GAG and collagen

To study the ability for fibrochondrogenic differentiation, the cell/scaffold constructs were incubated in fibrochondrogenic medium, as follows: α-minimum essential medium (α-MEM); Gibco BRL Co. Ltd., CTGF (100 ng/mL; PeproTech) and TGF-β3 (10 ng/mL; PeproTech), fibrogenic induction supplement [ascorbic acid (50 μg/mL)] and chondrogenic induction supplement [0.1 μM dexamethasone, sodium pyruvate (100 μg/mL), L-ascorbic acid 2-phosphate (50 μg/mL), L-proline (40 μg/mL), and 1% 1× insulin transferrin selenium (ITS)]. The DNA, glycosaminoglycan (GAG), and collagen in the cell/scaffold constructs were quantified after 7, 14, and 21 days of incubation, as described in our previous report 21. Briefly, the cell/scaffold constructs were digested for 16 h with a pre-prepared papain solution (125 mg/mL papain, 5 mM L-cysteine, 100 mM Na_2_HPO_4_, 5 mM EDTA, pH 6.2) (Sigma, St. Louis, MO, USA) at 60°C for DNA and GAG estimation. The 20 μL specimen digested above was reacted with 200 μL of Hoechst 33258 working solution (2 μg/mL) shielded from light at 37°C for 1 h. The intensity was measured by fluorimetry on a plate reader with an excitation of 360 nm and emission of 460 nm. The readings were compared with the standard curves of calf thymus DNA (Sigma). The total sulfated GAG content was estimated by the dimethylmethylene blue (DMMB, Sigma) assay. Next, 20 μl of sample digested as above was mixed with 200 μl of DMMB reagent and reacted for 30 min at room temperature, and the absorbance was measured on a plate reader at 525 nm. The content of GAG was calculated against a standard curve obtained from chondroitin 6-sulfate from shark (Sigma, USA). Type I and type II collagen contents were determined quantitatively by an enzyme-linked immunosorbent assay (ELISA) kit (Chondrex Inc., WA, USA) following the protocol.

#### Gene expression analysis *in vitro*

Quantitative RT-PCR was performed with SYBR Green PCR Master Mix (Toyobo, Osaka, Japan) to determine the expression of specific genes (collagen I, collagen II, sox 9, and aggrecan) using an ABI 7300 RT-PCR system (Applied Biosystems, Foster City, CA, USA) as previously described 22. In brief, the specimens were washed twice in PBS and lysed in TRIzol (Invitrogen). Total RNA was isolated and reverse transcribed to cDNA using a High-capacity cDNA Reverse Transcription kit (Applied Biosystems). The conditions of the RT-PCR were as follows: 95°C for 2 min, followed by 40 cycles of 95°C for 15 s and 60°C for 1 min. The 18s RNA was used as the housekeeping gene. The target genes were quantified by normalizing their expression to that of 18s RNA using the ΔΔCt method (the primers involved were listed in **Table S3**).

#### SMSCs SMSC recruitment *in vivo*

To assess the ability of the PS-L7 scaffold to recruit SMSCs *in vivo*, the PS and PS-L7 scaffolds were harvested 1 week after implantation in the knee-joint of the rat. Immunofluorescence staining was performed to assay the SMSC recruitment capacity of the two scaffolds. CD29 and CD90 were defined as MSC markers to distinguish the SMSCs. Briefly, the scaffolds were fixed in 4% paraformaldehyde at room temperature for 30 min and blocked by 10% goat serum for 1 h at room temperature. Subsequently, the samples were incubated with primary antibodies against CD29 (Proteintech, 12594-1-AP, 1:200) and CD90 (Abcam, ab225, 1:200) at 4°C overnight, followed by incubation with secondary antibodies for 1 h and Hoechst33258 (2 μg/mL; Fanbo, Beijing, China) for 10 min. The samples were observed by confocal microscopy (Leica, Nussloch, Germany) to determine the total cell numbers and CD29 and CD90 doubly labeled cell numbers.

### Animal experiments

The study animals were purchased from Peking University Animal Administration Center, and the experiment was performed under a protocol (LA 2016-263) approved by the Institutional Animal Care and Use Committee of Peking University, complying with the guidelines for the Care and Use of Laboratory Animals (National Academies Press, National Institutes of Health Publication No. 85-23, revised 1996). All animals were 3-month old New Zealand white rabbits weighing 2.7-3.2 kg, randomly allocated into three groups as follows: a control group with meniscectomy only and no graft implant; a PS group with meniscectomy and PS scaffold implant; and a PS-L7 group with meniscectomy and PS-L7 scaffold implant. The surgical procedure was performed as previously described (**Figure 1D**) 23. Briefly, the rabbits were anesthetized intravenously with 10 mL ethyl carbamate (0.2 g/mL) during the operation. After skin disinfection and draping, an anteromedial parapatellar incision was made, and the medial collateral ligament was cut down to expose the medial meniscus. Total medial meniscectomies were performed on both knees and the test groups were implanted with the appropriate scaffolds. The body of the implant was sutured to the capsule of the original meniscal rim, and the anterior and posterior horns were fixed by the tibial bony tunnel. The medial collateral ligament was sutured, and the wound was closed in layers. The animals received antibiotic prophylaxis with 10 mg/kg penicillin for 3 days postoperatively. No joint immobilization method was used after surgery. At each time point, the randomly selected animals were sacrificed via pentobarbital sodium.

#### Evaluation of meniscus

At each time point, the femoral condyles and the tibial plateaus with the implants were observed and photographed. The Gross Evaluation of Meniscus Implant Score was used to evaluate the implants, which has been reported in previous studies 10, 24, 25.

Briefly, the parameters were as follows: implant integration, implant position, horn position, shape, presence of tears in the implant, implant surface, implant size, tissue quality, and condition of the synovia. In this system, each parameter was scored from 1 to 3 based on the condition of the meniscal implants. The regenerated meniscal tissue was fixed in 4% paraformaldehyde for 48 h and then dehydrated and embedded in paraffin. Serial sections (6 mm thick) were prepared and stained with hematoxylin and eosin (H&E), toluidine blue (TB), Safranin O-Fast Green (SO), picrosirius red (PR) and immunohistochemistry of collagen I and II (Calbiochem). The meniscal histological score was used to analyze the sections of implants as in the previous study 24.

For each implant (total of 6 implants for each group), the inner, intermediate, and outer zones were evaluated. The blocks of implants were scored as follows: the presence of residual scaffold, foreign body response, cellularity, blood vessel ingrowth, fibrosis, cartilaginous matrix, integration of the implant with the joint capsule, and inflammatory cell infiltrate. The collagen contents of implants were determined semiquantitatively to determine the differences by immunohistochemical analysis using an Olympus BX-51 microscope and Image-Pro Plus 8.0 software (Media Cybernetics). The relative density (IOD/area) was measured to semi-quantify the deposition of collagen I and II.

#### Evaluation of cartilage

The articular cartilage was evaluated using the International Cartilage Repair Society (ICRS) cartilage lesion classification 26. The corresponding femoral condyle and tibial plateau were fixed in 4% paraformaldehyde for 48 h, then decalcified in 20% EDTA (pH 7.2) for one week. The samples were dehydrated and embedded in paraffin. The 6 mm thick serial sections were prepared for staining with TB or SO following the recommended protocol and graded according to the Mankins score system 27.

#### Scanning Electron Microscopy of Cartilage

To assess the cartilage surface microscopically, samples were observed by high-resolution SEM at 12 weeks and 24 weeks. The specimens were washed in PBS and fixed in 4 mL of 25% glutaraldehyde for 1 day at 4°C, dehydrated in a graded ethanol series and then dehydrated. The surface of the cartilage was vacuum-coated with a 5-nm layer of gold and then viewed using an SEM (S-2500; Hitachi High Technologies Co., Hitachi-Naka City, Japan).

#### Determination of inflammatory response

To determine whether there was an inflammatory response, the synovium in the knees were harvested and stained with H&E at 1, 3, and 6 weeks post-surgery. The synovial fluid was collected using a 2 mL syringe with an 18-gauge needle and centrifuged at 4000 rpm for 20 min at 4°C. The supernatants were collected and frozen at -80°C. Interleukin-1 (IL-1) and tumor necrosis factor-α (TNF-α) were assayed using standard ELISAs (Rabbit IL-1 ELISA Kit, SEA071Rb; Rabbit TNF-α ELISA Kit, SEA133Rb; Cloud-Clone Corp., USA).

#### Biomechanical analysis

The implants at 12 and 24 weeks, and the native meniscus were analyzed for biomechanical properties. The compression modulus of the implants was measured using nanoindentation as described previously 23. Samples were assayed using the Tri-boIndenter (Hysitron Inc., Minneapolis, Minnesota, USA) with a 20 μm 90° conical probe tip. For each measurement, 500 nm was defined as the maximum indentation depth, and a trapezoidal load was applied to each indentation site with loading (10 s), hold (2 s), and unloading (10 s). The microscopic geomorphology of the indentation zones was captured using a microscanning apparatus. The tensile test was performed by a material testing machine (AG-IS; Shimadzu) as previously described 28. The samples were cut into rectangular shapes from the peripheral edge of each meniscus.

### Statistical analysis

All data were expressed as the mean ± standard deviation. Statistical analyses were performed using SPSS 22.0 software. Significance values were determined by one-way analysis of variance (ANOVA) or the Mann-Whitney test, and *p* < 0.05 was considered statistically significant.

## Results

### Fabrication and characterization of scaffolds

In this study, the biomimetic wedge-shaped tissue-engineered meniscal scaffold with specific pore size and spatial structure was constructed using the 3DP technique 10. For the convenience of model preparation, the measured anatomical parameters were standardized. The scaffolds were “Roman Colosseum”-like structures, the edge-to-inner edge was a "concave arc", and the half of it, the ring at 180 ° was the meniscus model (**Figure S1**). According to the above parameters, the meniscal model was prepared with the SolidWork software, and the model was imported into the software system of a 3D-Bioplotter (EnvisionTEC 4th generation) printer, and “slide” layer cutting was performed. The interval time was 30 s, and the PCL scaffold was printed according to the set conditions. The PCL scaffold was composed of a ring-shaped fiber bundle and a radiation fiber bundle perpendicular to it, simulating the fiber direction of a natural meniscus. Subsequently, the SF solution was infused into the PCL scaffold and crosslinked (**Figure 1A**).

### Crosslinking of scaffolds

To avoid the cytotoxicity of residual compounds and overcome the effects of the traditional cross-linking method of SF, a double crosslink method (γ radiation and ethanol process) was employed (**Figure 1A**), which enhanced the elastic property, biodegradability, and biocompatibility of SF 29. In the γ-radiation phase, water absorbed energy from γ-rays to produce hydroxyl radicals (·OH), proton radicals (·H), hydrated electrons, and superoxide (O-_2_) (**Figure 3A**). The hydroxyl radicals activated SF molecules by attacking the SF molecular chain and extracting hydrogen from the polypeptide chain to yield large number of free radicals. Then, a covalent crosslinking reaction occurred between the free radicals and produced a uniform 3D SF hydrogel network. A similar reaction principle has been reported previously 30. The subsequent ethanol treatment promoted the transformation of SF secondary structure from α-helix to β-fold on the original 3D network (**Figure 3B**) 31. The pre-created γ-crosslinking network not only provided a preliminary supporting structure for the material system, but also affected the distribution and size of the new physical cross-linker β sheet domains, which contributed to the strength, elasticity, and stability of the SF sponge 32.

The scaffold materials were characterized by FTIR spectroscopy to determine the structural changes. The characteristic peaks of random coils in SF are shown as the 1650 cm^-1^ and 1534 cm^-1^ peaks in **Figure 3D** (1). It has been reported that γ-radiation does not cause changes in the secondary structure of silk 16. As shown in Fig. 3d (2) and (3), the 1650 cm^-1^ peak shifted to 1620 cm^-1^, and the 1534 cm^-1^ peak shifted to 1517 cm^-1^ after ethanol treatment. Corresponding to the β-fold structure of noncovalent cross-linking in SF, the characteristic peaks at 1620 cm^-1^ and 1517 cm^-1^ represented the tensile vibration of C=O in the Amide I interval and the deformation vibration of N-H in the Amide II interval, respectively, indicating the SF structural transition from random coils to a more stable β-fold structure 33. In the FTIR of untreated PCL shown in **Figure 3D** (4), the 1725 cm^-1^ peak represented the stretching vibration peak (C=O) of the carbonyl group, the 1245 cm^-1^ represented the asymmetric C-O-C stretching 34. As for the treated composite scaffold in **Figure 3D** (3), both of the characteristic peaks mentioned above also appeared. There was no significant change in the peak position, indicating that PCL was present in the composite scaffold, and the cross-linking procedure did not affect the structure of PCL.

### Microstructure of scaffolds

The PCL bundles were alternately oriented along the circumferential and perpendicular direction, mimicking the meniscal collagen alignment (**Figure 1A**). A macroscopic view of the different scaffolds is shown in **Figure 2A**, and SEM images in **Figure 2B** exhibit the PS scaffold intertwined SF micropores and PCL macropores forming an improved rough and broad surface of the scaffold (**Figure 2A-B**), which favored cell adhesion, retention, and differentiation.

### Degradation *in vitro*

As displayed in **Figure 2A**, the PS scaffolds showed unique profiles of degradation. Different from the slow degradation of PCL and the fast degradation of SF scaffolds, the degradation ratio of PS samples was between PCL and SF groups, greatly balancing the biomechanical properties and degradation rate to match the newly formed meniscus.

### Elasticity and frictional force of interface

To analyze the possible chondroprotective effect, a frictional force of interface of the two kinds of scaffolds and the compressive elasticity testing of SF scaffold was performed. The frictional force of the interface of PCL and SF scaffolds was 252.037 ± 11.087 Pa vs. 38.785 ± 10.918 Pa in PBS, and 195.176 ± 24.630 Pa vs. 4.418 ± 0.603 Pa in hyaluronic acid (**Figure 2D**). The compressive elasticity testing revealed that the SF sponge scaffold possessed the capability of recovering its original shape after being submitted to approximately 80% compressive strain (**Figure 2G**). These results indicated that SF sponge at the surface of the scaffold could reduce the interface friction between the implant and corresponding femoral condyle and tibial plateau and acted as a cushion for joint stress.

### Biomechanical properties of scaffolds

Compressive and tensile strength testing was performed to evaluate the biomechanical properties of the three different scaffolds. The compressive modulus of the SF, PCL, and PCL-SF groups was 0.081 ± 0.028 MPa, 5.689 ± 0.760 MPa, and 6.582 ± 0.645 MPa, respectively (**Figure 2E**). The tensile modulus of the three scaffolds was 0.336 ± 0.142 MPa, 11.067 ± 3.363 MPa, and 13.402 ± 3.119 MPa, respectively (**Figure 2F**). Therefore, the biomechanical properties of PS scaffold were superior to those of the other two pure scaffolds (both in terms of compressive and tensile strength).

### Bioactivity of scaffolds conjugated to affinity peptide

To improve the SMSC-specific recruitment ability for better meniscus regeneration, an SMSC affinity peptide L7 (LTHPRWP), which was identified by phage display technology described in our previous report 14, was conjugated to PS scaffolds. After peptide conjugation, confocal scanning microscopy exhibited homogeneous green fluorescence indicative of conjugation of the L7 peptide on the PS scaffold surface (**Figure S2**). A cell recruitment analysis was performed to evaluate the SMSC affinity bioactivity of L7 peptide conjugated to the scaffolds both *in vitro* and *in vivo*. In the *in vitro* experiment, the effluent number of cells was higher in the PS scaffold than the PS-L7 scaffold after 12 and 24 h of culturing, indicating that the conjugation of L7 enhanced the SMSC recruitment capacity of scaffolds (**Figure 4C**). The recruitment capacity of L7 *in vivo* was also verified. Previous studies have reported that MSCs highly express CD29 and CD90 35, 36. Here, as shown by immunofluorescence results, the* in vivo* SMSC recruitment capacity of the functionalized scaffold was superior to that in the PS scaffold, with significantly more CD29^+^/CD90^+^ cells in the PS-L7 group than in the PS group (**Figure 4B**). These results revealed that the conjugation procedure did not compromise the bioactivity of L7 peptide, which enhanced the ability of the scaffold to recruit SMSCs.

### Biocompatibility analysis of scaffolds

To evaluate the biocompatibility of scaffolds, we analyzed the viability, morphology, proliferation, and chondrogenic properties of SMSCs by the two kinds of scaffolds. After incubation on the scaffolds for 3 days, cell viability and morphology were investigated by confocal microscopy and the results showed that SMSCs grew well on both scaffolds, with no obviously dead cells (**Figure 4Aii-iii**). The alamarBlue assay further verified favorable cell viability on both scaffolds at day 1, 3, 5, and 7 (**Figure 4D**). After fibrochondrogenic incubation for 21 days, glycosaminoglycan (GAG), collagen type I (COL I) and collagen type II (COL II) production were measured and normalized by DNA content to quantify fibrocartilaginous matrix production. On day 21, the PS-L7 group had significantly more deposition of GAG, COL I, and COL II than the PS group (**Figure 4E-G**). Also, fibrogenic (*Col1*) and chondrogenic gene expressions (*Col2*, *Sox9,* and *Acan*) were significantly upregulated in both groups at all time points (**Figure 4H-K**). These results demonstrated that the scaffolds could provide an ideal platform for the growth, proliferation, and ECM secretion of SMSCs.

### Evaluation of meniscus regeneration *in vivo*

**Figure 5:**
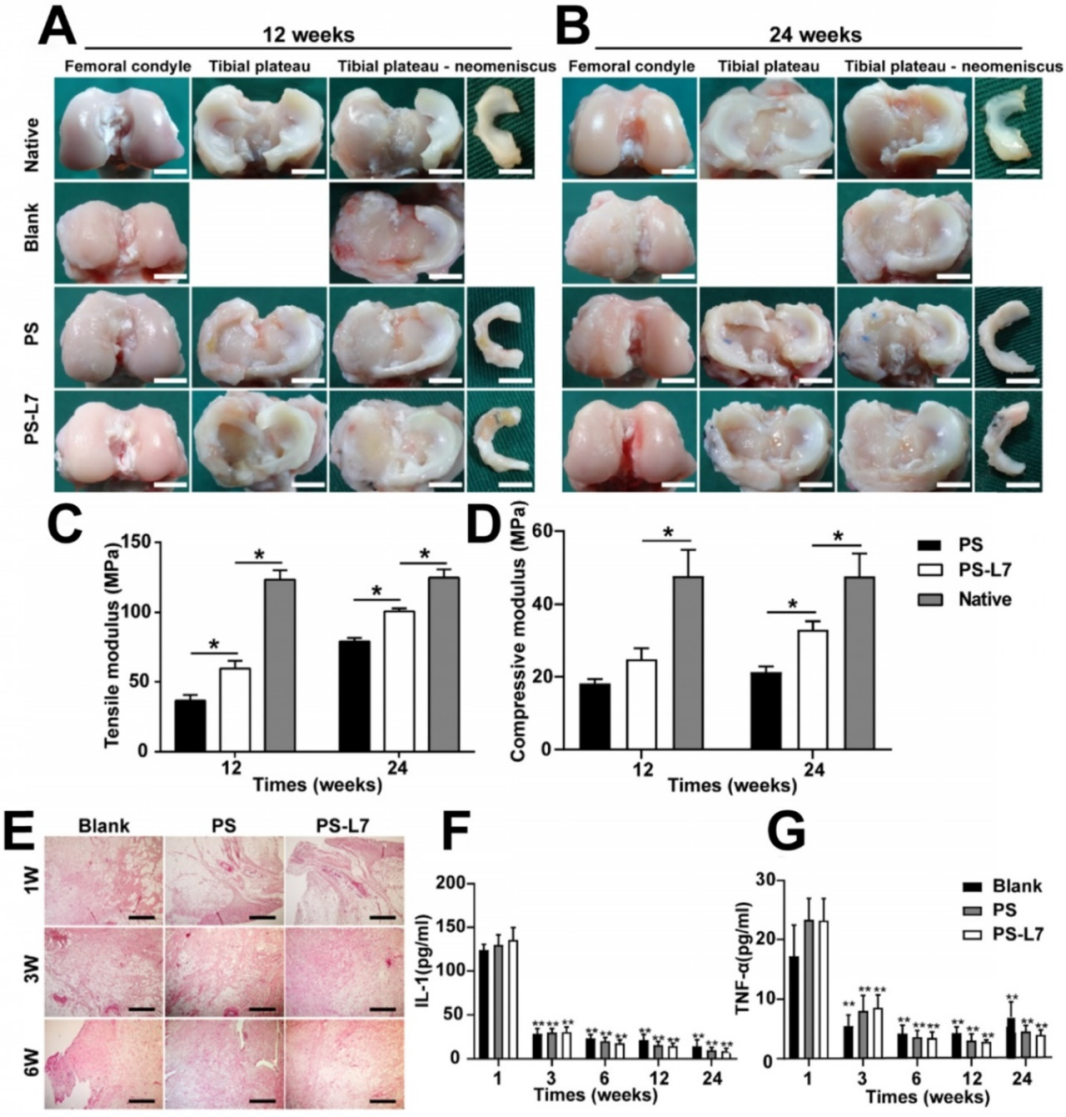
Macroscopic observation, biomechanical and inflammation assessment of regenerated meniscus *in vivo*. (A-B) Macroscopic observation of joints at 12 weeks and 24 weeks after implantation. (scale bar = 10 mm) Medial meniscal excised from the tibial plateau is shown on the right. The Blank group received no implantation after total medial meniscectomy. (C-D) Biomechanical assay of implants at each time point (12 weeks and 24 weeks) (n = 4, *p < 0.05). (E) Histological evidence of the synovium at 1, 3, 6 weeks after surgery (scale bar = 200 µm). (F) Quantitative assay of Interleukin-1 in the synovial fluid at 1, 3, 6, 12, and 24 weeks after surgery. (G) Quantitative assay of tumor necrosis factor-α in the synovial fluid at 1, 3, 6, 12, and 24 weeks after surgery. (n = 6, **p < 0.01 vs 1 week)

The general morphology and histological features of implants were assessed to analyze the meniscus regeneration. Gross observation of regenerated meniscus is displayed in **Figure 5A-B**. There was no obvious meniscal tissue regeneration in the Blank group. Compared with the PS scaffold, the PS-L7 scaffold had a more complete and uniformly regenerated meniscus, better horn position, and tissue strength, and was better integrated to the joint at 12 weeks post-surgery (**Table S1**). The total score between the two groups showed a significant statistical difference (*p* < 0.05). At 24 weeks post-operation, the neo-meniscus of the PS-L7 group exhibited superior performance in all aspects of the Kon E Gross score.

**Figure 6:**
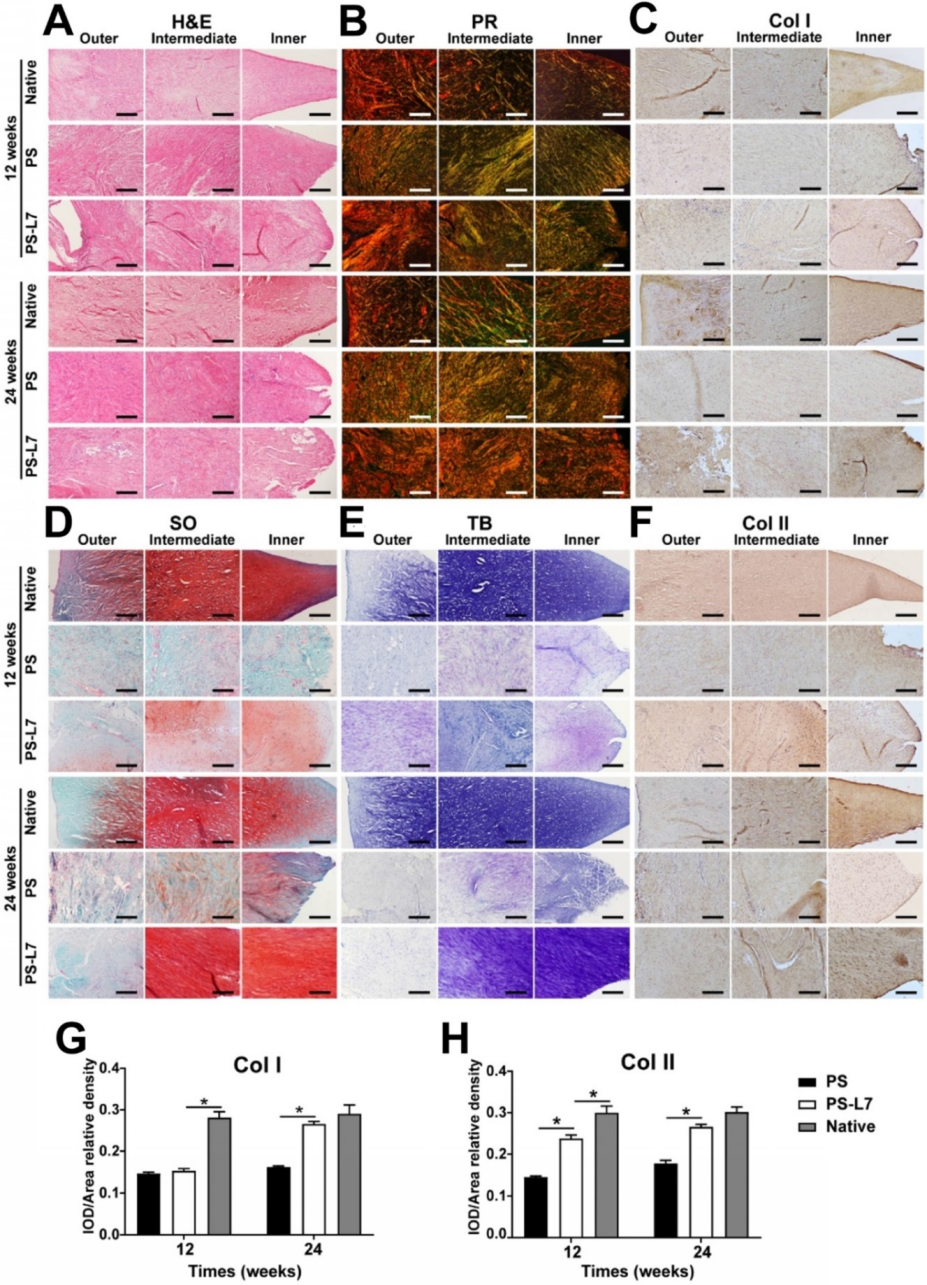
** Histological assessment of outer, intermediate, and inner zones of implants and native meniscus *in vivo*. (A)** H&E staining; (B) PR staining; (C) Immunohistochemical staining for Col I; (D) SO staining; (E) TB staining, (F) Immunohistochemical staining for Col II. (G-H) Immunohistochemical semiquantitative analyses of native meniscus and implants at 12 and 24 weeks after surgery. Values for integrated optical density (IOD) per area of (G) Col I and (H) Col II were larger in the PS-L7 group compared with the PS group, similar to the native meniscus at 24 weeks. (scale bar = 200 µm) (n = 5, *p < 0.05)

The histological features (**Figure 6A**, **Table S2**) of implants showed no obvious inflammatory cell infiltration in all groups. Residual scaffolds were observed in the PS and PS-L7 groups at both 12 weeks and 24 weeks. Compared with the PS group, the PS-L7 group revealed relatively low foreign body reaction and few hypocellular areas at both time points. All groups exhibited new blood vessels, especially in the synovial edge of the neo-meniscus. Besides, new cartilaginous matrix deposition in the PS-L7 group had better performance than the PS group. PR, TB, SO, and immunohistochemistry staining were conducted to determine the collagen and proteoglycan content of implants. PR staining (**Figure 6B**) showed COL I in the PS and PS-L7 groups to be similar at 12 weeks. Then the PS-L7 group increased more noticeably than the PS group in the outer region of the implant at 24 weeks, demonstrating a well-organized arrangement of collagen. These results were consistent with the results of COL I immunohistochemistry (**Figure 6C**). SO (**Figure 6D**) and TB (**Figure 6E**) staining showed identical results for proteoglycans, which were better in the PS-L7 group than in the PS group at each time point, and were distributed in the intermediate and inner regions of the implants as was evident from COL II immunohistochemistry (**Figure 6F**). Moreover, a semi-quantitative analysis of COL I and II (**Figure 6G-H**) was used to evaluate the collagen content. The IOD/area relative density of COL I and II in two groups was less than the native meniscus at 12 weeks. The PS-L7 group increased greatly at 24 weeks and was closer to the native meniscus. The expression of COL I and II in the PS-L7 group was higher than in the PS group at 24 weeks. These results suggested that, compared to the control scaffolds, the scaffolds conjugated by L7 promoted superior meniscus regeneration, which was closer to the native meniscus.

### Evaluation of joint cartilage degeneration

**Figure 7:**
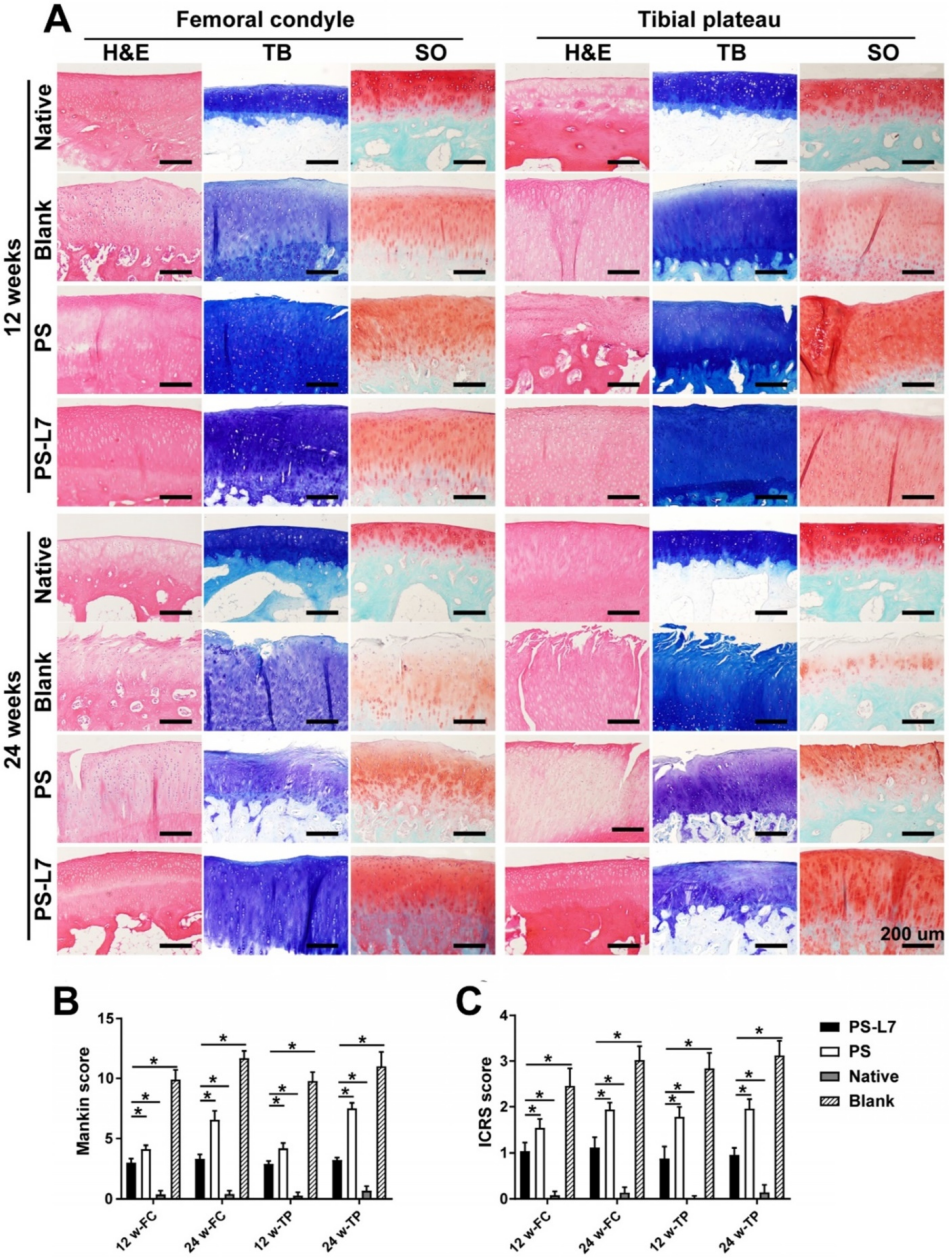
** Histological assessment of cartilage* in vivo*.** (A) Histological assessment (H&E, TB, and SO staining) of the femoral condyle and tibial plateau cartilage in different groups at 12 weeks and 24 weeks. (B) International Cartilage Repair Society (ICRS) and Mankin scores of articular cartilage surfaces in the femoral condyle and tibial plateau; the PS-L7 group exhibited lower cartilage degeneration in both the femur and tibia compared with the PS group or Blank group. (scale bar = 200 µm) (n = 5, *p < 0.05)

**Figure 8:**
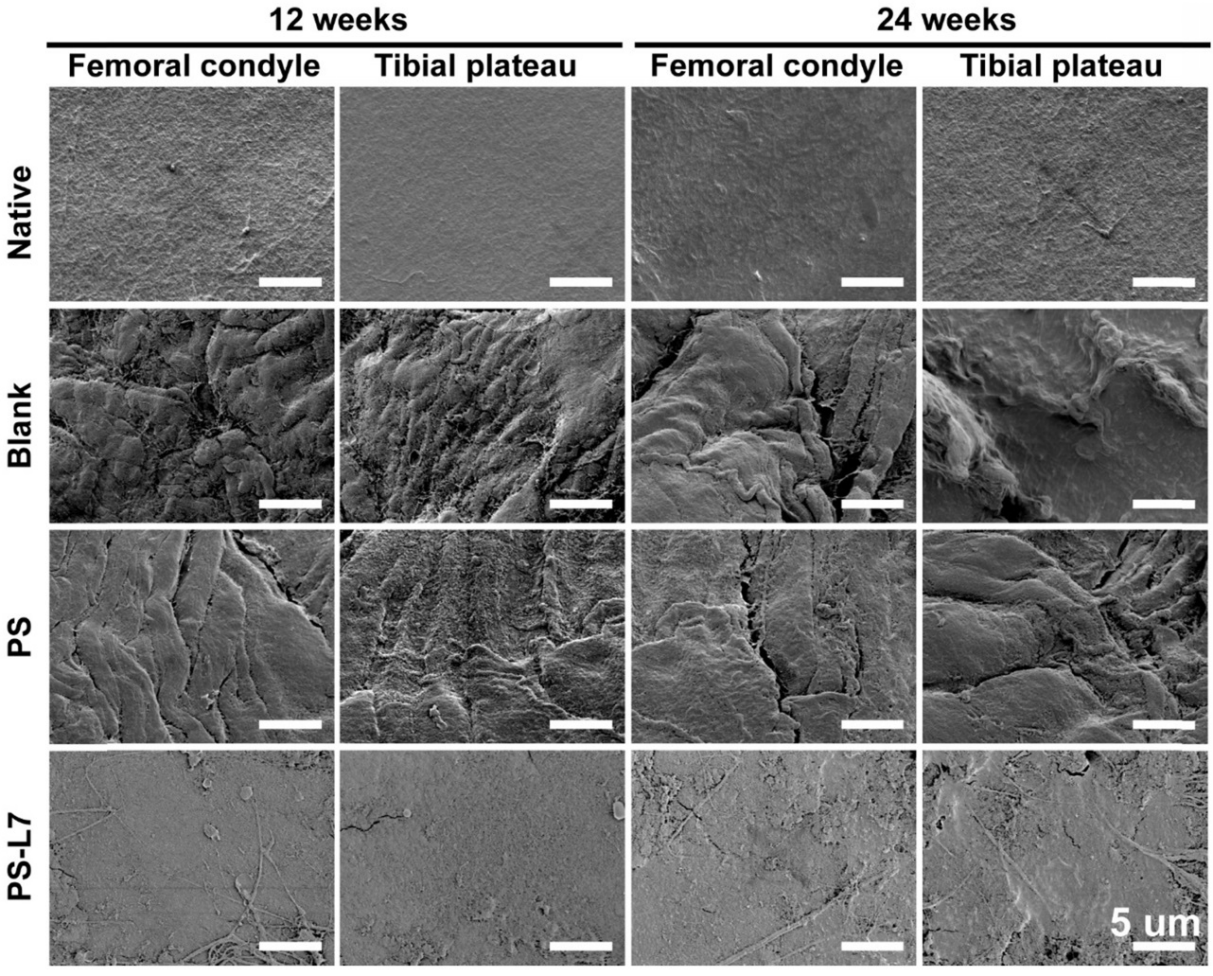
** SEM images of the femoral condyle and tibial plateau cartilage in different groups at 12 and 24 weeks.** (scale bar = 5 µm)

It was important to evaluate the chondroprotective effect of various scaffolds. Gross observation of cartilage (**Figure 5A-B**) showed less cartilage degeneration in the PS-L7 group than in PS and Blank groups at each time point, which was also verified by histological analysis (**Figure 7A**). ICRS and Mankins scores showed the worst cartilage performance progressing with time in the Blank group followed by the PS group (**Figure 7B-C**). HE, TB, and SO staining showed obvious cartilage degradation and clefts in the Blank and PS groups but seldom in the PS-L7 group, demonstrating a better cartilage protection ability of the PS-L7 group. Also, SEM results of the femoral condyle and tibial plateau revealed a similar change in histology (**Figure 8**). Overall, the Blank group showed deep cracks and lacunae in the femoral condyle and tibial plateau. The PS group also exhibited superficial cracks and tangled fibrous surfaces, while in the PS-L7 group, the cartilage revealed flatter surface with scarcely any cracks.

### Inflammation response

To check whether the scaffolds would cause significant rejection and foreign body reactions, we evaluated the synovium and synovial fluid. Synovial histologic staining (**Figure 5E**) and quantification results of inflammatory factors IL-1 and TNF-α (**Figure 5F-G**) showed that the inflammation response initially increased at week 1 (acute period), then declined, and finally remained at a low level (week 3, 6, 12 and 24).

### Biomechanical properties of implants

The biomechanical properties of regenerated meniscus were analyzed at 12 and 24 weeks (**Figure 5C-D**). At 12 weeks, the compressive and tensile moduli of implants of both PS and PS-L7 scaffolds were inferior to that in the native meniscus. At 24 weeks, however, the compressive and tensile moduli in the PS-L7 group was stronger than those of the PS group, showing higher similarity to the native meniscus.

## Discussion

In this study, we constructed a functionally modified tissue-engineered scaffold of SF and PCL with the L7 peptide for meniscus regeneration. As a natural biomaterial with advantages of low immunity and good biocompatibility for cell infiltration and proliferation, SF has been widely used in meniscus repair 6, 37, 38. However, biomechanical properties of SF are far inferior to those of the native meniscus. To overcome this problem, we used a synthetic polymer PCL with remarkable 3D printability and biomechanical properties 10, A biomimetic wedge-shaped scaffold with specific pore size and the spatial structure was constructed employing 3DP that matched the need of meniscus tissue engineering scaffolds. The 3DP technique is a promising method for clinical applications because it can be customized for each patient to address the problems of size-matching and individualization 11.

The PCL bundles were alternately oriented along the circumferential and perpendicular direction, mimicking the meniscal collagen alignment (**Figure 1A**). When the meniscus was subjected to extruding outward and downward pressure during the motion of the knee, the circumferential and radial PCL fiber bundles and the tensile strength fixed at the anterior and posterior horns could mimic the natural meniscus in terms of resisting stress load and avoiding injury and dislocation of the reconstructed meniscus. Besides, the SF sponge with fine elasticity and low interfacial shear force also enhanced the energy absorption capacity of the meniscus, improving chondroprotection (**Figure 2D, G**). The addition of PCL significantly improved the biomechanical properties of the scaffold, as shown by biomechanical testing *in vitro* (**Figure 2E-F**).

Before then, several investigators have attempted to use PCL scaffolds to emulate the architecture and function of native meniscus. Chen et al. constructed a hybrid meniscus scaffold by 3D printing of a wedge-shaped PCL scaffold as a backbone followed by injection with the optimized MECM-based hydrogel 39. Mandal and co-workers fabricated knee meniscus grafts of a three-layered wedge-shaped silk meniscal scaffold system to mimic the native meniscus architecture 6. In another study, Gao et al. combined the decellularized meniscus extracellular matrix (DMECM) with PCL via electrospinning to fabricate a meniscus scaffold 40. In our study, the compressive modulus of the PS-L7 scaffold was similar to that in Chen's study, which was better than that of the multilayered silk scaffold reported by Mandal et al. Also, the tensile modulus of PS-L7 scaffold we formulated was higher than that of PCL/DMECM scaffold investigated by Gao's group. These results showed that our scaffold had a better performance than the existing scaffolds with respect to biomechanical properties. The combination of SF and PCL could greatly balance the biomechanical properties and degradation rate to match the requirement of meniscus tissue engineering scaffolds, as was evident by the degradation profile *in vitro* (**Figure 2C**). To avoid the cytotoxicity of residual compounds and overcome the limitations of the traditional crosslinking method, a double crosslinking method (γ radiation and ethanol process) was employed, which enhanced the elastic property, biodegradability, and biocompatibility of SF sponge 29.

Another key factor for tissue engineering is cellular adhesion and retention onto the scaffold. In our study, SF solution was infused into the PCL scaffold and crosslinked to yield intertwined SF micropores and PCL macropores hence forming an improved rough and broad surface of the scaffold (**Figure 2A-B**), which favored cell adhesion, retention, and differentiation. It has been reported that, after meniscus injury, surrounding SMSCs migrate to the injured site, proliferate, and differentiate into fibrochondrocytes under the influence of local microenvironment 41, 42. It has also been shown that SMSCs are a promising cell source for meniscus regeneration with a similar gene expression profile as the meniscus cells 43-46. In the present study, following the fabrication of the meticulously designed scaffold, a proven SMSC-specific affinity peptide L7 was conjugated onto the scaffold to improve SMSC recruitment, as we previously described 14. The superior SMSC recruitment ability of the PS-L7 scaffold demonstrated that the conjugation process did not compromise the bioactivity of the peptide, and the cell viability and proliferation behavior assays indicated no cytotoxic residue. The cytocompatibility assay demonstrated that the scaffolds could provide an ideal platform for the growth, proliferation, and differentiation of SMSCs.

In the *in vivo* studies, the macroscopic view revealed better meniscus regeneration in the PS-L7 group than in the PS group (**Figure 5A-B**) due to the favorable cell recruitment and differentiation. Significantly more cell infiltration and production of GAG, outer regional COL I, and inner regional COL II were observed in the PS-L7 group by H&E (**Figure 6A**), PR (**Figure 6B**), SO (**Figure 6D**), TB (**Figure 6E**), and immunohistochemistry staining (**Figure 6C, F**).

The cartilaginous histological evidence of the femoral condyle and tibial plateau revealed cartilage degradation and clefts in the Blank group, which were less severe in the PS group and rare in the PS-L7 group (**Figure 7A**), demonstrating that the PS-L7 group provided the best chondroprotective effect among all groups. The PS-L7 group had a statistically significant higher Gross and Histological Evaluation of Meniscal Implant Score (**Table S1, S2**), and lower International Cartilage Repair Society (ICRS) and Mankins score (**Figure 7B-C**) than the PS group, and exhibited anisotropic architecture similar to the native meniscus, indicating better meniscus regeneration and chondroprotective effect.

Furthermore, there was no indication of inflammation or rejection reactions in the synovial fluid and synovium (**Figure 5E-G**) caused by either of the scaffolds supporting their suitability for meniscus repair. The biomechanical properties of regenerated implants (**Figure 5C-D**) in the PS-L7 group were superior to those in the PS group, demonstrating better tissue ingrowth ability and a more orderly arrangement and distribution of collagen fibers, which were more conducive to bearing load and resisting stress. The biomechanical analysis of implants *in vivo* showed that the compressive and tensile moduli of the PS-L7 scaffold were similar to the native meniscus. Thus, the structural and biomechanical optimization afforded a tissue engineering scaffold as an ideal option for load-bearing and maintenance of biomechanical microenvironment.

## Conclusion

In this study, we developed a meticulously tailored PCL/SF scaffold augmented by SMSC affinity peptide L7 using 3DP technology with excellent structural, biomechanical, and functional properties for meniscus regeneration. With the advantages of biomimetic architecture, SMSC recruitment ability, and excellent biomechanical characteristics, the scaffold provided an excellent microenvironment for SMSC recruitment, retention, proliferation, differentiation, and ECM production. Furthermore, the scaffold displayed superior biomechanical properties and excellent anisotropic meniscus regeneration and chondroprotection. Compared with traditional cell-based therapies, the current study provides a novel approach for one-step meniscus repair and regeneration with the advantages of reduced cost and avoiding secondary operation. Thus, the PS-L7 scaffold developed in the current study exhibits tremendous potential for clinical translation in meniscus tissue engineering.

## Supplementary Material

Supplementary figures and tables.
