# Supplementary material for: Biomechanically, structurally and functionally meticulously tailored polycaprolactone/silk fibroin scaffold for meniscus regeneration
Source: Theranostics. 2020 Apr 6;10(11):5090–106. doi: 10.7150/thno.44270 (PMC7163455; doi:10.7150/thno.44270)
Supplement: Supplementary file 1 — Supplementary figures and tables. [file thnov10p5090s1.pdf]

Supplementary data

Biomechanically, structurally and functionally meticulously tailored polycaprolactone/silk fibroin scaffold for meniscus regeneration

Zong Li[1*], Nier Wu[2*], Jin Cheng[1*], Muyang Sun[1], Peng Yang[1], Fengyuan Zhao[1], Jiahao Zhang[1], Xiaoning Duan[1], Xin Fu[1], Jiying Zhang[1], Xiaoqing Hu[1✉], Haifeng Chen[2✉] and Yingfang Ao[1✉]

1. Institute of Sports Medicine, Beijing Key Laboratory of Sports Injuries, Peking University Third Hospital, 49 North Garden Road, Haidian District, Beijing 100191, People's Republic of China
2. Department of Biomedical Engineering, College of Engineering, Peking University, 5 Yiheyuan Road, Haidian District, Beijing 100871, People's Republic of China

* Contributed equally to this work

✉ Corresponding author: Yingfang Ao, E-mail: aoyingfang@163.com, Phone: （010） 82267390, Fax: 86-10-62010440. Haifeng Chen, E-mail: haifeng.chen@pku.edu.cn. Xiaoqing Hu, E-mail: huxiaoqingbd01@sina.com

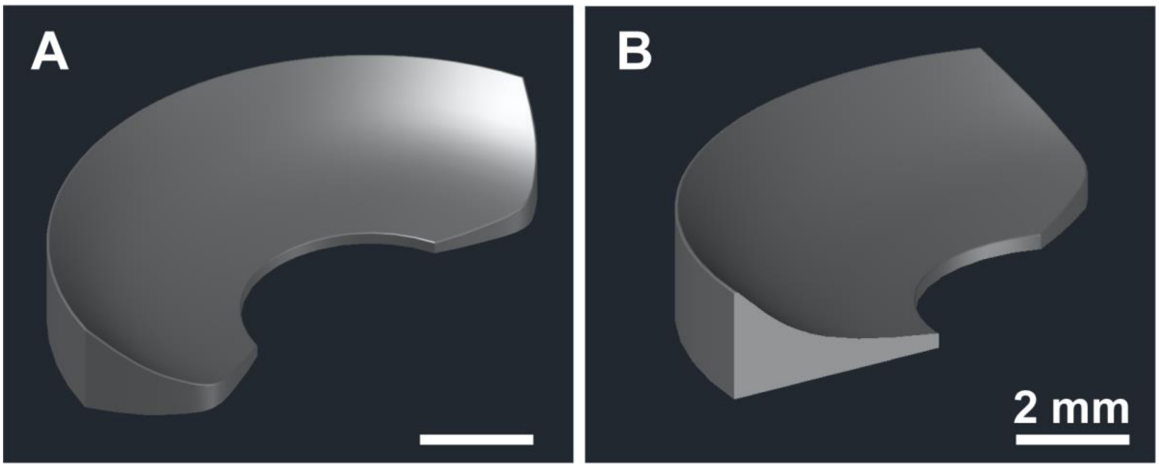

Figure S1. Meniscus model. (A) Top observation, **(B)** Lateral observation.

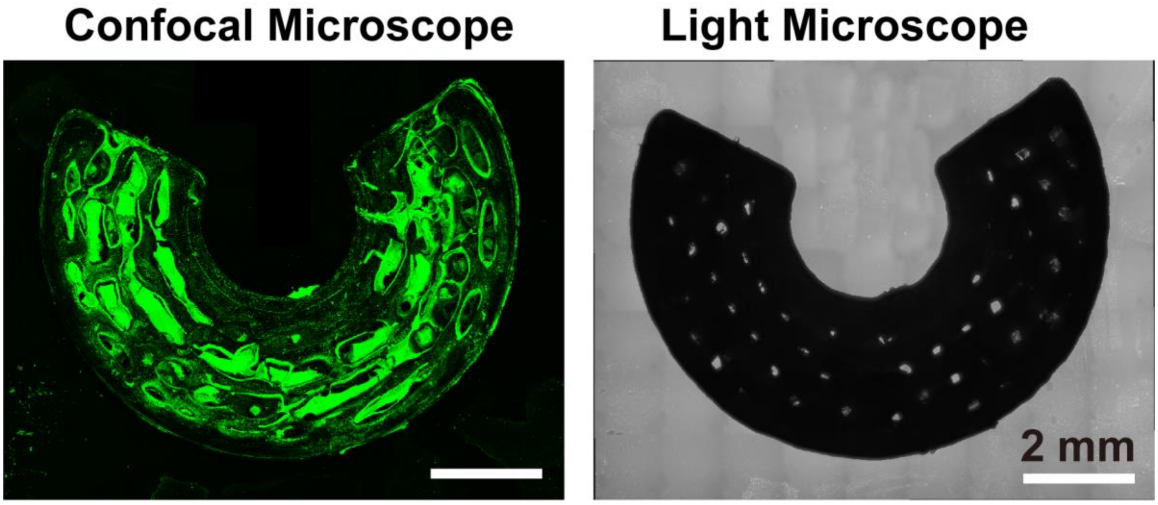

Figure S2. Confocal images of scaffold showed conjugation of FITC-L7 peptide.

Table S1. Gross Evaluation of Meniscal Implant Score[a]

Parameter	PS-12W	PS-L7-12W	PS-24W	PS-L7-24W	*P* value[b]	
					α	β
Integration	1.3(1-2)	2.1(1-3)	1.7(1-2)	2.3(2-3)	0.001*	0.012*
Implant position	1.6(1-2)	2.0(1-3)	1.7(1-2)	2.2(2-3)	0.047*	0.012*
Horn position	1.6(1-3)	2.3(2-3)	1.8(1-3)	2.4(2-3)	0.004*	0.021*
Shape	1.7(1-2)	2.1(1-3)	1.6(1-2)	2.8(2-3)	0.094	0.001*
Tears	2.3(2-3)	2.6(2-3)	2.4(2-3)	2.8(2-3)	0.101	0.008*
Surface	1.2(1-3)	1.5(1-2)	1.8(1-3)	2.4(2-3)	0.101	0.000*
Size	1.4(1-2)	1.7(1-2)	1.8(1-3)	2.4(2-3)	0.101	0.013*
Tissue strength	1.9(1-3)	2.3(2-3)	2.0(1-3)	2.5(2-3)	0.047*	0.004*
Synovia	2.1(1-3)	2.4(2-3)	2.1(1-3)	2.5(2-3)	0.060	0.047*
Total score	15.1(10-23)	19.0(13-25)	16.9(10-24)	22.3(18-27)	0.000*	0.000*

[a]Each parameter is scored from 1 to 3 based on the condition of the meniscal implant. Scores are provided as mean (range).
[b]α represents PS-12W vs PS-L7-12W; β represents PS-24W vs PS-L7-24W.
* represents statistically significant (*$p < 0.05$).

Table S2. Histological Features of Implants[a]

Feature	PS-12W	PS-L7-12W	PS-24W	PS-L7-24W
Residual scaffold	5 (100)	5 (100)	5 (100)	5 (100)
Foreign body reaction	5 (100)	4 (80)	5 (100)	3 (60)
Hypocelluar areas	5 (100)	4 (80)	5 (100)	3 (60)
Blood vessels	5 (100)	5 (100)	5 (100)	5 (100)
Fibrosis	5 (100)	4 (80)	3 (60)	2 (40)
Cartilage metaplasia				
Tip	3 (60)	4 (80)	4 (80)	5 (100)
Central	2 (40)	3 (60)	3 (60)	5 (100)
Integration				
Good	3 (60)	4 (80)	4 (80)	5 (100)
Poor	2 (40)	1 (20)	1 (20)	0 (0)
Inflammatory infiltrate				
Lymphocytes	5 (100)	5 (100)	5 (100)	4 (80)
Plasma	0	0	0	0
Neutrophils	0	0	0	0

[a]The features were scored as being either present or absent for each implant (total of 5 implants for each group). Data are provided as n (%).

Table S3. Real-time PCR primers

Gene	Forward primer (5'–3')	Reverse primer (5'–3')
Type I collagen	gCAATgCTgAATCgTCCCAC	CAgCACAggCCCTCAAAAAC
Type II collagen	CACCgCTAACgTCCAgATgAC	ggAAggCgTgAggTCTTCTgT
Sox9	AgCTgTgTgTAgACgggTTg	TCCCCgCAACAgATCTCCTA
Aggrecan	CATTCgCACgggAgCAgCCA	TggggTCCgTgggCTCACAA
18s RNA	gTAACCCgTTgAACCCCATT	CCATCCAATCggTAgTAgCg
